# Variations in levels of IL-6 and TNF-α in type 2 diabetes mellitus between rural and urban Ashanti Region of Ghana

**DOI:** 10.1186/s12902-015-0047-9

**Published:** 2015-09-21

**Authors:** Samuel N. Darko, Denis D. Yar, Ellis Owusu-Dabo, Anthony Afum-Adjei Awuah, Williams Dapaah, Nicholas Addofoh, Samson P. Salifu, Nana Y. Awua-Boateng, Fred Adomako-Boateng

**Affiliations:** Kumasi Centre for Collaborative Research in Tropical Medicine, Kwame Nkrumah University of Science and Technology (KNUST), Kumasi, Ghana; School of Public Health, KNUST, Kumasi, Ghana; Department of Biochemistry and Biotechnology, KNUST, Kumasi, Ghana; Ghana Health Service, Kumasi, Ashanti Region Ghana

## Abstract

**Background:**

A surge in pro-inflammatory markers, Il-6 and TNF-α, has been associated with type 2 diabetes mellitus (T2DM). However, there is no data on the dynamics of these markers in T2DM Ghanaian populations. The aim of this study was to determine variations in the levels of IL-6 and TNF-α in T2DM patients. This study also examined the associations of IL-6 and TNF-α with anthropometric measurement and the effect of co-morbidity with hypertension using rural and urban dwellers in the Ashanti region, Ghana.

**Methods:**

A nested case–control design using participants aged 25–70 years consisting of 77 T2DM ± hypertension patients and 112 controls were selected from a larger study on Research on Obesity and Diabetes among African Migrants (RODAM). Anthropometric measurements, blood pressure and body fat percentage were measured. Fasting blood samples were analyzed for glucose, IL-6 and TNF-α levels.

**Results:**

The median level of IL-6 was significantly higher (*p* < 0.0001) among rural dwellers compared to urban dwellers. Inversely, urban dwellers had significantly higher (*p* = 0.0424) median level of TNF-α compared to rural cases. No significant differences were observed in IL-6 (*p* = 0.3571) and TNF-α (*p* = 0.2581) among T2DM patients compared with T2DM ± hypertension patients. A weak negative correlation was found between IL-6 and BMI in urban T2DM.

**Discussion:**

The average level of IL-6 was higher in rural T2DM participants compared with those in urban setting. However, higher levels of TNF-α was observed among the study participants with T2DM in urban settings compared to those of rural.﻿ In this study, we observed that co-morbidity of hypertension had no significant effect on the levels of IL-6 and TNF-α. We are of the opinion that higher physical activity levels among rural particpants and high obesity levels in urabn participants explain the observation but needs more numbers to validate.

**Conclusion:**

This study revealed that IL-6 levels were higher among rural dwellers than urban while TNF-α levels were higher in urban dwellers than rural in patients with T2DM. There was no association of body fat percentage and body mass index with IL-6 and TNF-α levels. Co-morbidity of hypertension with T2DM had no effect on IL-6 and TNF-α levels.

## Background

Non-communicable diseases (NCDs) are currently a burden for the global health system [1–3]. Previous studies have indicated that sub-Saharan Africa has the highest incidence rate of type 2 diabetes mellitus (T2DM) which is projected to double from 12 million to 24 million by 2030 [4, 5]. Low chronic inflammation associated with T2DM has fueled investigations into pro-inflammatory biomarkers as early indicators for the detection of T2DM to curtail its progression and associated morbidities [6–9]. However, there is a knowledge gap on the effect of co-morbidities such as hypertension and obesity, which are precursors for low-grade chronic inflammation. Also, differences in environmental settings could affect the levels of pro-inflammatory markers among T2DM patients from an African population.

Previous studies conducted to assess variations in inflammatory markers associated with type 2 diabetes in urban populations have indicated a significant difference in the levels of interleukin 6 (IL-6) and tumour necrosis factor α (TNF-α) [10, 11]. Nested case–control studies conducted mainly among Caucasian, Asian and specific native populations showed IL-6 and TNF-α as predictors for T2DM with varied correlations with anthropometric measurements [7, 8, 11]. High prevalence of obesity in urban settings could cause an increase in pro-inflammatory cytokines. IL-6 and TNF-α have been observed to have a positive association with obesity in urban dwellers [12]. Most of these studies conducted on inflammatory markers associated T2DM have predominantly sampled urban populations [10, 13] without comparing the effect of lifestyle and environmental differences on these marker levels in rural settings of same geographical areas. As a result, little is known about variations in pro-inflammatory markers between urban and rural dwellers with T2DM and compared with healthy populations in both settings. Moreover, to date, no study has been conducted on the dynamics of IL-6 and TNF-α among the T2DM Ghanaian population.

Hence, in this study, we assessed the variations in the levels of, IL-6 and TNF-α in T2DM patients, associations between anthropometric measurement and these pro-inflammatory markers and the effect of co-morbidity with hypertension among rural and urban dwellers in the Ashanti region, Ghana.

## Methods

### Participants

A nested case–control study using data from a larger study; Research on Obesity and Diabetes among African Migrants (RODAM) [14]. The study data was collected between 2012 and 2014 in a population consisting of Ghanaians aged 25 – 70 years. The urban population was recruited from communities in the Kumasi Metropolis (the second most densely populated city in Ghana) and Obuasi Municipality, while the rural participants were from villages in the same region (Ashanti). The procedure for recruiting and interviewing of participants has been published [14].

### Sample size and study power

Sample size of 189 was estimated using the Cochran method with an assumed T2DM prevalence of 14.4 % in Ashanti region, Ghana at 95 % confidence level nested into cases and controls for both rural and urban settings assuming a default study power of 80 % with α of 5 %.

### Selection criteria

A case was defined as participant diagnosed with fasting plasma glucose of ≥ 7.0 mmol/L and plasma glucose value of ≥ 11.1 mmol/L, 2 h after a 75 g oral glucose tolerance test [15] with or without hypertension. A control for the study was a participant with fasting plasma glucose ≤ 7.0 mmol/L without hypertension recruited in the study sites. Participants were selected by simple random selection.

Body weight was measured to the nearest 0.1 kg with a digital scale (SECA 877, UK) after removal of footwear, heavy clothing and pocket contents. Height to the nearest 0.1 m was measured using a portable stadiometer (SECA 217, UK). Body Mass Index (BMI) was computed as weight in kg divided by the square of the height in meters (kg/m^2^). Using appropriate cuff sizes, three blood pressure readings were taken on the left arm with the Microlife^®^ WatchBP^®^ (UK) in a seated position with at least 15 min rest. The mean of the three readings was used. Hypertension was defined as blood pressure of 140/90 mmHg or more [16]. Body fat% was estimated using Bioimpedance and analysis with an impedance analyzer (BODYSTAT^®^ 1500 MDD, UK).

Written informed consent was obtained from each participant for inclusion for the study while non-consenting individuals and pregnant women were excluded from the study. This study was approved by the Committee for Human Research Publications and Ethics at the School of Medical Sciences, Kwame Nkrumah University of Science and Technology and the Komfo Anokye Teaching Hospital, Kumasi.

### Laboratory methods

Fasting venous blood samples were collected from each participant by a phlebotomist at all study sites. Blood samples collected were manually processed and aliquoted immediately according to standard operational procedures and cryopreserved before transporting to laboratory for analysis as previously described [14]. Fasting blood glucose was determined using the glucose dehydrogenase method with Accu-Chek® Perfoma Analyzer (Roche, Germany) with a coefficient of variation of less than 5 % compared to a laboratory chemistry analyzer. Serum samples were analyzed for IL-6 and TNF-α using highly sensitive sandwich ELISA. IL-6 was measured in blood serum with a highly sensitive sandwich ELISA using Human IL-6 ELISA test kit (BIOO SCIENTIFIC, USA) with minimum detectable concentration of 8 pg/mL [17]. TNF-α levels were determined using sandwich ELISA test kit with a minimum detection concentration of 12 pg/mL [18].

### Statistical analysis

Data were entered into Microsoft Excel and statistical analysis performed using Graphpad Prism 5 software. Data normality was tested using D’Agostino and Pearson omnibus normality test. The statistical difference between medians was estimated by the Mann–Whitney test. Associations were measured by coefficients of Spearman’s partial correlation whiles controlling for age and gender. Differences were considered statistically significant at *p* < 0.05.

## Results

### Median levels of pro-inflammatory markers within rural and urban settings

The median levels of TNF-α were 258.5 (32.4 – 514.5) in urban T2DM and 188.0 (52.5 – 412.0) in rural T2DM participants (Table 1). There were no differences in TNF-α level between cases and controls within the same setting (Table 1). In the urban population, the median level of IL-6 was elevated among the control group [43.9 (0.9 – 716.2)] relative to the T2DM cases (*p* = 0.8327). However, IL-6 levels among the rural population in T2DM cases were significantly higher than in the controls (*p* value < 0.0001).

**Table 1:** Characteristics of participants and laboratory investigations

Variables	Median (range)/n (%)		*p* value
	T2DM Cases	Controls	
Urban	n = 37	n = 42	
Age (years)	50 (27–69)	45.5 (31–70)	0.3477
Sex			
Male	8 (22)	5 (12)	
Female	29 (78)	37 (88)	
Height (m)	1.6 (1.5 – 1.8)	1.6 (1.4 – 1.8)	0.0057^a^
Weight (kg)	66.4 (44.5 - 96.6)	62.5 (34.1 - 93.3)	0.1615
BMI (kg/m^2^)	27.3 (20.0 - 38.7)	26.4 (17.7 - 48.4)	0.1341
Body Fat (%)	48.3 (25.9 - 66.5)	48.0 (23.8 - 61.2)	0.9569
Hypertension	9 (23)	NA	
FBS (mmol/L)	7.5 (7.0 - 18.8)	5.7 (4.7 - 6.6)	<0.0001^a^
IL-6 (pg/mL)	38.7 (12.3 - 129.3)	43.9 (8.0 - 500.0)	0.8327
TNF-α (pg/mL)	258.5 (32.4 - 514.5)	158.8 (14.18 - 6320)	0.0714
Rural	n = 40	n = 70	
Age (years)	51.5 (28–70)	49 (25–70)	0.3429
Sex			
Male	19 (48)	29 (41)	
Female	21 (52)	41 (59)	
Height (m)	1.6 (1.5 – 1.2)	1.6 (1.2 – 1.8)	0.2796
Weight (kg)	59.70 (43.35 - 78.6)	58.1 (36.4 - 101.8)	0.7047
BMI (kg/m^2^)	21.57 (9.64 - 30.5)	20.68 (15.8 - 33.4)	0.5571
Body Fat (%)	42.9 (26.3 - 60.28)	43.1 (10.7 - 60.4)	0.8254
Hypertension	19 (51)	NA	
FBS (mmol/L)	7.5 (7.0 - 13.7)	6.0 (4.6 - 6.9)	<0.0001^a^
IL-6 (pg/mL)	108.4 (26.0 - 478.0)	48.3 (4.8 - 432.8)	<0.0001^a^
TNF- α (pg/mL)	188.0 (52.5 - 412.0)	184.5 (53.3 - 700)	0.5282

T2DM: type 2 diabetes mellitus, BMI: body mass index, FBS: fasting blood sugar, IL-6: interleukin-6, TNF- α: tumour necrosis factor-alpha. NA: not applicable

^a^statistically significant

### Median levels of pro-inflammatory markers in T2DM cases between rural and urban settings

**Fig. 1:**
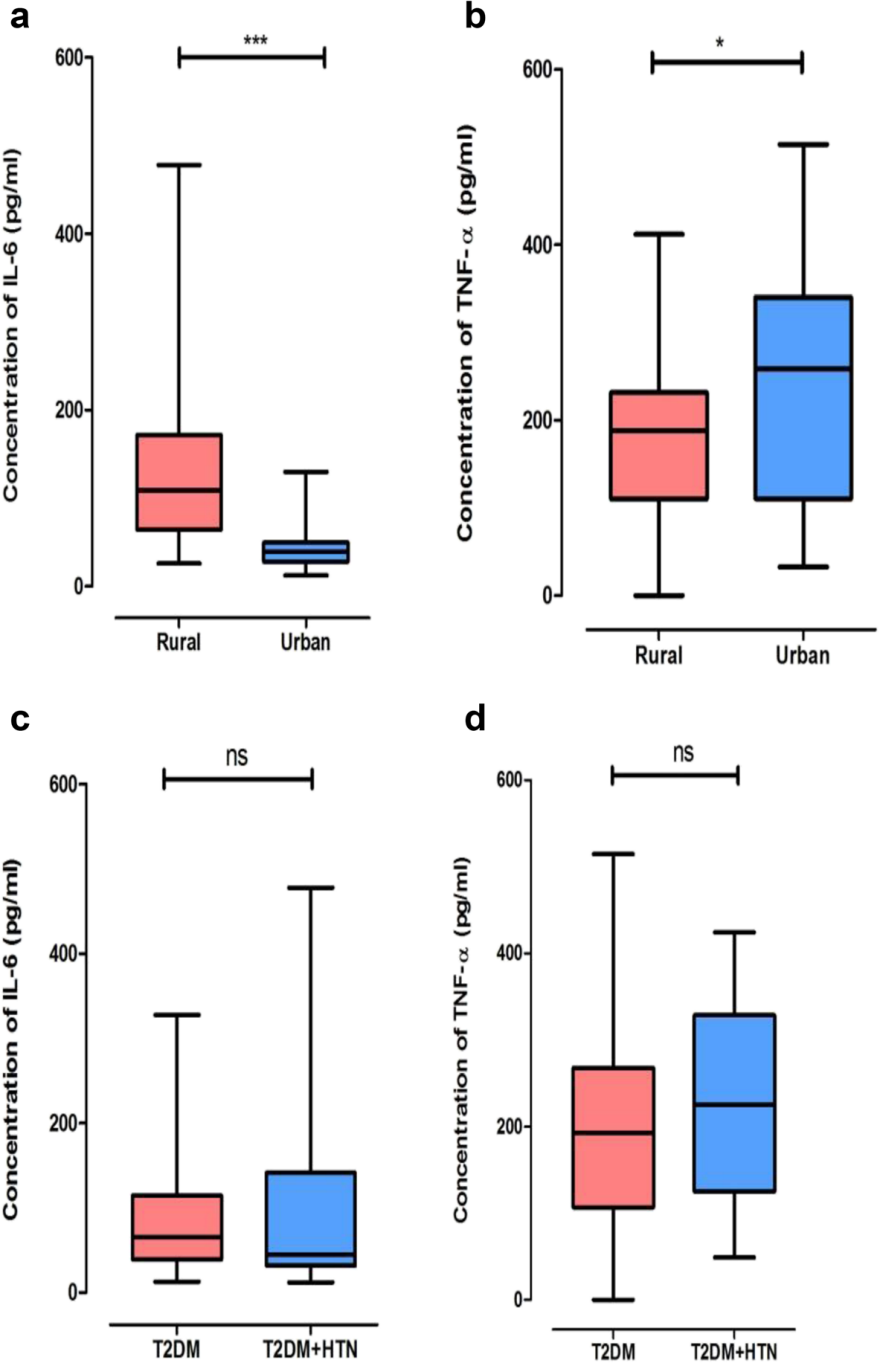
Variations in levels of pro-inflammatory markers and the effect of hypertension between settings. *p = 0.0424, ***p < 0.0001, ns = not statistically significant, T2DM + HTN = type 2 diabetes mellitus + hypertension. **a** Overall levels of IL-6 among rural and urban participants. **b** Overall levels of TNF-α among rural and urban participants. **c** Levels of IL-6 among rural and urban participants with only T2DM compared with T2DM and Hypertension. **d** Levels of TNF-α among rural and urban participants with both T2DM and Hypertension

IL-6 levels were significantly higher (*p* < 0.0001) among the rural cases compared to those of the urban cases (Fig. 1a). Inversely, urban cases had a significantly higher (*p* = 0.0424) median level of TNF-α compared to rural cases (Fig. 1b).

### Measures of adiposity and fasting blood sugar between rural and urban settings

Both urban and rural dwellers had median age of 50 years (Table 2) with ages ranging from 27 to 70 and 25–70 respectively (*p* = 0.8270). There were more female participants in the urban (84 %) setting compared to rural (56 %) area. Both BMI and body fat percentage were significantly higher in the urban participants compared with those in rural setting (*p* values of < 0.0001 and 0.0002 respectively).

**Table 2:** Measures of adiposity and fasting blood sugar between urban and rural settings

Variables	Median (range)/n (%)		*p* value
	Urban	Rural	
	n = 79	n = 110	
Age	50 (27–70)	50 (25–70)	0.827
Age Category (years)			
25-34	6 (7.59)	19 (17.27)	
35-44	24 (30.38)	23 (20.91)	
45-54	27 (34.18)	31 (28.18)	
55-64	16 (20.35)	27 (24.55)	
65+	6 (7.59)	10 (9.09)	
Sex			
Male	13 (16)	48 (44)	
Female	66 (84)	62 (56)	
BMI	27.2 (17.7 - 48.4)	20.9 (15.0 - 33.4)	<0.0001
BMI Category (kg/m2)			
BMI < 18.5	4 (5.07)	20 (18.18)	
18.5 ≤ BMI ≤ 24.9	25 (31.65)	66 (60.00)	
25.0 ≤ BMI ≤ 29.9	27 (34.18)	18 (16.36)	
BMI ≥ 30	23 (29.11)	6 (5.45)	
Body Fat%	48.1 (23.8 - 66.5)	43.1 (10.7 - 60.4)	0.0002
FBS	6.5 (4.7 - 18.8)	6.6 (4.6 - 13.7)	0.9400
FBS Category			
FBS < 6.0	29 (36.71)	30 (27.27)	
6.0 ≤ FBS ≤ 6.9	13 (16.46)	40 (36.36)	
FBS ≥ 7.0	37 (46.84)	40 (36.36)	

BMI = body mass index, FBS = fasting blood sugar

### Effect of co-morbidity of hypertension on levels of IL-6 and TNF-α

There was no association observed in IL-6 (*p* = 0.3571) and TNF-α (*p* = 0.2581) levels in patients with only T2DM compared with patients with T2DM + HTN (Figs. 1c and 1d).

### Associations between anthropometric and pro-inflammatory markers in T2DM

IL-6 was negatively but weakly correlated with BMI (r = −0.3765, *p* = 0.0258) in the urban study group (Table 3).

**Table 3:** Associations between anthropometric and pro-inflammatory markers in T2DM

	r (*p* value)	
Setting	IL-6	TNF- α
Rural		
BMI	0.1094 (0.5131)	0.0698 (0.6773)
Body Fat %	0.0719 (0.6679)	−0.0251 (0.8813)
Urban		
BMI	−0.3765 (0.0258)	0.1243 (0.4769)
Body Fat %	0.0467 (0.7901)	0.0374 (0.8312)

Adjusted for age and gender

BMI: body mass index, FBS: fasting blood sugar, IL-6: interleukin-6, TNF- α: tumour necrosis factor-alpha, r: Spearman’s partial correlation coefficient

## Discussion

There are very limited studies conducted in Ghana to assess the correlation of IL-6 and TNF-α among T2DM patients with hypertension and non-diabetic participants in rural and urban settings. Our results have showed varying levels of IL-6 and TNF-α between T2DM cases and controls in both rural and urban settings. The average level of IL-6 was higher in rural T2DM participants compared with those in urban setting. However, higher levels of TNF-α was observed among the study participants with T2DM in urban settings compared to those of rural. In this study, we observed that co-morbidity of hypertension had no significant effect on the levels of IL-6 and TNF-α. To the best of our knowledge, this is the first time a study of this kind has been conducted in Ghana while only limited related studies have been conducted in Africa [19]. The high level of IL-6 observed among T2DM cases from the rural setting is fairly similar to what was found in previous studies conducted by Krakoff and Nadeem [7, 10] although these studies were conducted in a native Pima Indians in Mexico and Pakistani populations respectively. However, in a related study by Al-Shukaili et al. (2013) [13] among an urban Asian population, patients with T2DM had decreased levels of IL-6 compared to healthy controls. While a cross-sectional study by Hossain and colleagues found no significant difference in serum levels of IL-6 between pre-diabetic participants and controls [20]. The difference between our findings and these other studies may be attributed to several reasons, including differences in case groups, varying sample sizes, gender composition, differences in environmental and social settings and the duration of the T2DM disease. The significantly higher levels of IL-6 among the rural T2DM compared to urban population observed in our study, we suspect could result from the synergistic effect of pro-inflammatory action of IL-6 in T2DM and its anti-inflammatory myokine action [21] induced by physical exercise. Circulating IL-6 has been found to increase to about 100 fold in response to physical activity and this is dependent on the intensity, duration and endurance capacity [22–24] of the individuals. We suspect that, the more physical and active lifestyle of rural dwellers may likely predispose them to produce an increase levels of IL-6 more than the relatively sedentary lifestyle among urban dwellers. Lifestyle variations between rural and urban dwellers could possibly have contributed to the marked differences in the observed levels of IL-6.

In this study, T2DM urban cases had a significantly higher average level of TNF-α compared to those of rural cases. It has been established that, TNF-α, is predominantly secreted from adipose tissues which are more among females [25]. In this study, majority of the study participants with T2DM was females and many were of urban settings. Females generally have more adipose tissues and body fat percentage with a consequential increase in body weight relative to their male counterparts. Hence, we suspect that the high TNF-α levels may have been contributed by the number of urban female participants with T2DM (Table 2). Various pro-inflammatory factors are known to be associated and linked with the risk of T2DM and obesity, a metabolic syndrome. However, differences in the socio-demographic characteristics result in variations in obesity among Ghanaian adults [26]. Consequently, this could explain for differences in the observed levels of TNF-α between the rural and urban populations. This assertion agrees with findings by Park et al. (2005) which showed a positive association of obesity and visceral adiposity with high serum concentrations of C-reactive protein, IL-6 and TNF-α [27]. Also, a similar study by Goyal et al. [12] found that obese diabetics had higher levels of TNF-α compared to non-obese diabetics [12].

There was no significant difference between the mean values of IL-6 and TNF-α among participants with T2DM and T2DM + HTN in both rural and urban settings. However, median values of IL-6 and TNF-α in T2DM + HTN group compared with T2DM group suggests that co-morbidity with hypertension may possibly have a cumulative effect. Previous studies have established that lesions due to pulmonary arterial hypertension could cause activation of macrophages which subsequently secretes several cytokines including IL-6 and TNF-α [28, 29]. However, this assertion could not be advanced in our study since our sample size was relatively small and could have contributed in the values obtained.

We found in this study that BMI and TNF-α had a positive but weak correlation for both rural and urban settings, though not significant, but consistent with findings from previous studies [12, 30] which found no significant association. This positive linear relationship could be attributed to an increase in the adipose macrophages responsible for secreting TNF-α and the associated increase in adipose cells among persons with high BMI. However, in this study, BMI had a significant but negatively weak correlation with IL-6 in the urban T2DM group contrary to earlier findings [30, 31], which showed positive correlation implicating increase in adiposity with a rise in the levels of adipokines. This difference in our findings with these previous studies could be attributed to differences in the study group compositions, racial, environmental and nutritional differences.

There were some inherent limitations associated with this study including our sample size. We had fewer males with T2DM relative to the females especially in the urban setting which may have influenced the results. Moreover, there are confounders such as physical activity, infections and auto-immune diseases that could influence the levels of these pro-inflammatory biomarkers. The findings in this study have shown that rural and urban settings could affect variations in IL-6 and TNF- α levels in T2DM population. Further investigations into these and other pro-inflammatory markers in specific populations is needed to ascertain their relevance both as early predictors and diagnostic cut offs for T2DM.

## Conclusion

We conclude there was higher level of IL-6 among rural type 2 diabetics compared to those in urban setting whiles higher TNF-α level in urban than rural was found in the Ashanti region of Ghana. There was no association between anthropometric measurement and IL-6 and TNF- α levels and co-morbidity of T2DM with hypertension had no effect on IL-6 and TNF- α levels.

## Availability of data and materials

Not applicable.

## Abbreviations

T2DM: Type 2 diabetes mellitus
IL-6: Interleukin-6
TNF- α: Tumuor necrosis factor-alpha
RODAM: Research on Obesity and Diabetes among African Migrants
NCDs: Non-communicable diseases
BMI: Body mass index
ELISA: Enzyme-linked immunosorbent assay
